# Depressive and Neurocognitive Disorders in the Context of the Inflammatory Background of COVID-19

**DOI:** 10.3390/life11101056

**Published:** 2021-10-08

**Authors:** Eliza Dąbrowska, Beata Galińska-Skok, Napoleon Waszkiewicz

**Affiliations:** Department of Psychiatry, Medical University of Bialystok, pl. Brodowicza 1, 16-070 Choroszcz, Poland; beata.galinska@umb.edu.pl (B.G.-S.); napoleon.waszkiewicz@umb.edu.pl (N.W.)

**Keywords:** COVID-19, SARS-CoV-2, neuroinflammation, depressive disorders, depression, neurocognitive disorders, post-covid, long-term, complications

## Abstract

The dysfunctional effects of the coronavirus disease 2019 (COVID-19) infection on the nervous system are established. The manifestation of neuropsychiatric symptoms during and after infection is influenced by the neuroinvasive and neurotrophic properties of SARS-CoV-2 as well as strong inflammation characterised by a specific “cytokine storm”. Research suggests that a strong immune response to a SARS-CoV-2 infection and psychological stressors related to the pandemic may cause chronic inflammatory processes in the body with elevated levels of inflammatory markers contributing to the intensification of neurodegenerative processes. It is suggested that neuroinflammation and associated central nervous system changes may significantly contribute to the etiopathogenesis of depressive disorders. In addition, symptoms after a COVID-19 infection may persist for up to several weeks after an acute infection as a post-COVID-19 syndrome. Moreover, previous knowledge indicates that among SSRI (selective serotonin reuptake inhibitor) group antidepressants, fluoxetine is a promising drug against COVID-19. In conclusion, further research, observation and broadening of the knowledge of the pathomechanism of a SARS-CoV-2 infection and the impact on potential complications are necessary. It is essential to continue research in order to assess the long-term neuropsychiatric effects in COVID-19 patients and to find new therapeutic strategies.

## 1. Introduction

At the time of writing this review, the entire world was struggling with the coronavirus disease 2019 (COVID-19) pandemic, which has affected nearly every aspect of society [1]. Despite the duration of the pandemic for many months, SARS-CoV-2 infection still hides many unknowns, especially regarding its long-term effects [2,3]. COVID-19 is an acute infectious respiratory disease caused by a novel virus named SARS-CoV-2. It is a single-stranded RNA virus with about 80% similarity to SARS-CoV from the coronavirus family [4]. The first cases of infection were reported at the turn of 2019/2020 in China. Rapid global transmission of the virus was observed with an increasing wave of infections and deaths. In March 2020, The World Health Organization (WHO) declared a COVID-19 pandemic [5]. As of 30 August 2021, the COVID-19 pandemic had over 216 million confirmed cases with 4.5 million deaths [6]. Along with the destabilisation of everyday life, negative psychological consequences of the pandemic and the emergence of mental disorders among societies have been observed [7,8,9]. In the course of a COVID-19 infection, in addition to typical respiratory symptoms, a dysfunctional effect of SARS-CoV-2 on other body systems, including the nervous system, has been noticed [10,11]. A range of COVID-19 neurological symptoms has been observed from mild symptoms such as a headache or dizziness to loss of taste, smell, meningitis and even necrotic encephalitis [12,13]. According to the latest scientific reports, the manifestation of neuropsychiatric symptoms during and after a COVID-19 infection is influenced by the neuroinvasive and neurotrophic properties of SARS-CoV-2 as well as by strong inflammation in the course of the infection characterised by a specific “cytokine storm” [14,15,16]. Potentially life-threatening cytokine storm syndrome is characteristic of severe COVID-19 [17]. Research suggests that a strong immune response to a SARS-CoV-2 infection and psychological stressors related to the pandemic may cause chronic inflammatory processes in the body with elevated levels of inflammatory markers contributing to the intensification of neurodegenerative processes and the emergence of other psychiatric complications [18,19,20,21]. Inflammation, related changes and damages at the level of the nervous system in the course of SARS-CoV-2 have been suggested to significantly contribute to the etiopathogenesis of depressive disorders [22,23,24]. Furthermore, studies show that some acute viral infections can induce an abnormal immune response, affecting the manifestation of long-term neuropsychiatric consequences [25,26]. Experiences of previous viral pandemics, such as the flu of the 18th and 19th centuries, Spanish flu in the 20th century and the coronaviruses causing severe acute respiratory syndrome (SARS) in 2002 and the Middle East respiratory syndrome (MERS) in 2012, suggest a link with the manifestation of neuropsychiatric complications both during and after an acute infection [27]. Among the survivors after infection with SARS-CoV and MERS coronaviruses, there have been cases of memory, attention and concentration disturbances lasting up to 39 months after the onset of the disease [28,29]. It has been suggested that the severity of neuropsychiatric complications correlates with the severity of COVID-19. It is observed that those patients, after experiencing severe COVID-19 with respiratory symptoms, leaving intensive care units are potentially more likely to experience long-term neuropsychiatric and neurocognitive conditions such as depression, obsessive-compulsive disorder, psychosis, Parkinson’s disease and Alzheimer’s disease [20,30]. The consequences of SARS-CoV-2 infection on the body have a wide and serious impact and are still being investigated [31]. In this review, we focus on the problem of depressive and neurocognitive disorders in the face of COVID-19, the pathogenesis of SARS-CoV-2 infection, its long-term consequences for the body and potential therapeutic interventions, including antidepressant treatment. The studies conducted so far indicate the immunomodulatory and antiviral properties of antidepressant treatment, which may appear effective in counteracting neuropsychiatric complications after COVID-19 [32,33].

## 2. Materials and Methods

Articles were searched from various databases, including PubMed, Google Scholar and MEDLINE, using the following keywords: SARS-CoV-2, COVID-19, cytokine storm, inflammation, neuroinfection, neuroinflammation, depressive disorders, neurocognitive disorders, post-COVID syndrome, antidepressants. The duration for viewing potentially interesting articles was from 1 December 2020 until 18 August 2021. Selected articles concerned the issues of inflammation in the course of COVID-19 and neuropsychiatric complications after COVID-19 infection. Moreover, publications on the effect of antidepressant treatment on COVID-19 were reviewed. The conclusions were drawn up on the basis of the available literature and our own reflections.

## 3. Inflammatory Basis of Depression

### 3.1. Depression as a Global Problem

Depressive disorders are a common and serious health problem. Depression is the leading cause of disability in the world, which is a personal and socioeconomic challenge [34]. Depression affects about 5–17% of the population and is one of the most common mental disorders in the society [34]. More than 350 million people worldwide suffer from depression, and about 40–80% of them have suicidal thoughts, while 20–40% attempt suicide, of which 12–18% are successful. Each year, it is estimated that about one million people worldwide die from depression [35,36]. The prevalence of depressive disorders in the 18–29 age group is 3 times higher than that of the age of 60 and more [37]. Women suffer from depression more often than men, and incidence rates increase in women with age. Beginning in early adolescence, the incidence rate in women is 1.5 to 3 times highthan in men [37].

### 3.2. The Concept of the Inflammatory Basis of Depression

**Figure 1:**
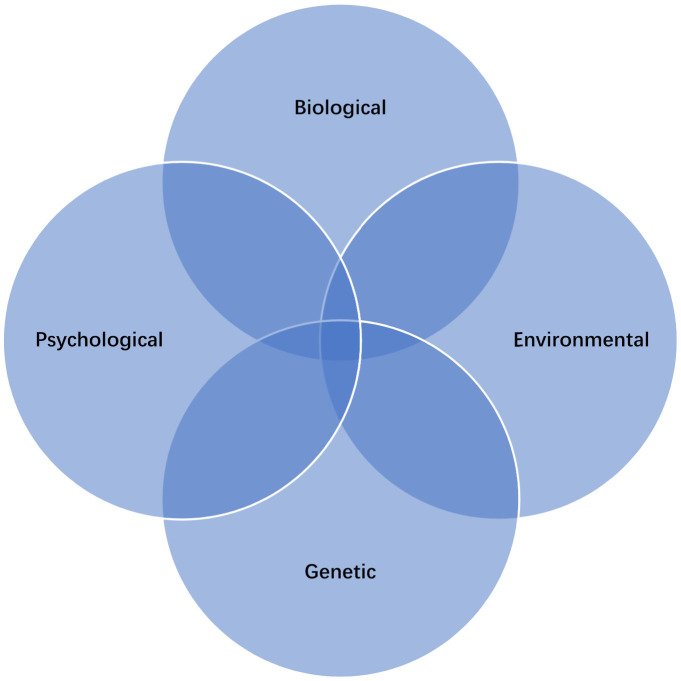
Multifactorial aetiology of depression.

The aetiology of depression is multifactorial, consisting of biological, genetic, psychological and environmental factors which, according to the neurodevelopmental theory, complement each other (Figure 1) [37,38,39]

In addition to the current main neurobiological concepts of depression—including the monoaminergic theory with brain neurotransmission dysfunction, the dysregulation of the hypothalamic–pituitary–adrenal (HPA) axis [40] and structural changes in the brain mainly in the hippocampus and frontal lobes [41,42]—the key role of inflammation in the nervous system in the development of depression is increasingly emphasised [43,44].

#### 3.2.1. The Hypothalamic–Pituitary–Adrenal Axis (HPA) and Role of Chronic Stress

The hypothalamic–pituitary–adrenal (HPA) axis as the stress axis is adaptive in order to maintain homeostasis by the body. It plays a key role in responding to stress stimuli, both physical and mental. As a result of the activation of the HPA axis, the sympathetic nervous system is stimulated with the release of cortisol and adrenaline. In the event of a prolonged stressful situation or impaired functioning of the feedback loop, chronic stress occurs [45,46,47]. Chronic stress is one of the strongest inhibitors of neurogenesis, leading to a decrease in the proliferation of neuronal stem cells and the survival of new nerve cells in the dentate gyrus of the hippocampus. As a result of stress stimulation of the hypothalamic–pituitary–adrenal axis, there is an increase in the amount of glucocorticosteroids adversely affecting neurogenesis and an increase in the expression of their receptors on the hippocampus. The secretion of stress hormones is potentiated by pro-inflammatory factors, including interleukin-1 (IL-1) [48,49]. Hypercortisolaemia with excessive autonomic activation has an adverse effect on the body, causing immunosuppression, increased blood pressure, increased cholesterol levels and the dysfunction of the production of sex hormones [45,50,51]. Moreover, the number of glucocorticosteroid receptors is lowered, and their sensitivity is reduced, which causes feedback loop disorders. In addition, the long-term activation of the HPA axis causes the influx of calcium ions to the hippocampal neurons, leading to their apoptosis. There are structural and functional changes in the hippocampus and a reduction in its volume due to the neurotoxic effect of hypercortisolaemia [45,52,53]. Recent studies show that chronic stress can lead to primary microglia activation by influencing the vessels [47,54]. As a result of chronic stress, there may also be a reduction in the level of neurotrophins and growth factors promoting neurogenesis and disturbances in the level of neurotransmitters, such as Gamma-aminobutyric acid (GABA), serotonin, glutamate, dopamine and noradrenaline, which are important in maintaining mental well-being [48]. Changes in the brain caused by a reaction to long-term or severe stress may appear with memory and learning difficulties as well as affecting the manifestation of affective disorders and depression [35,41,43]. In terms of pathophysiology, depressive disorders resemble chronic stress [55]. In the group of people with depression, an increased concentration of corticotropin releasing factor (CRF) in the cerebrospinal fluid and an increase in the secretion of the hypothalamic neurohormone arginine vasopressin (AVP) were both observed [56]. This is associated with an impaired pituitary response to stimulation by CRF as a result of receptor down-regulation for CRF of the anterior pituitary lobe. One study evaluating the pituitary response to CRF stimulation in women with/without an episode of major depression and/or with a history of sexual abuse with assessment of CRF and AVP concentrations in CSF showed nearly 60% variability in the ACTH response to CRF stimulation [56].

#### 3.2.2. Neuroinflammation and Brain Changes in Depression

In patients with depression, the presence of inflammation and activation of the immune system both in the periphery and in the central nervous system is found [57]. There is a diminished T-lymphocyte profiling response and over-activation of the non-specific inflammatory response and the acute-phase inflammatory response mechanisms [58]. There is an increase in oxidative stress and lipid peroxidation and a decrease in the activity of antioxidant enzymes, with an increase in the production of reactive oxygen species [59,60]. The results of the studies indicate the presence of elevated levels of acute phase proteins, including C-reactive protein (CRP), haptoglobin, α1-antitrypsin (A1AT) and Alpha-1-acid glycoprotein (AGP), in some depressed people (who do not develop the inflammatory process of a known aetiology) [61]. Studies also indicate the presence of inflammatory mediators such as prostaglandin E (PGE2), a pro-inflammatory nitric oxide-NO molecule [62,63,64]. In addition, prolonged and severe depressive states may be accompanied by increased plasma levels of pro-inflammatory cytokines IL-1, IL-2, a soluble IL-2 receptor (sIL-2R), tumour necrosis factor α (TNF-α), IL-8, IL-18 and interferon- γ (IFN-γ) and a decrease in acute phase protein concentrations of albumin and transferrin as well as IL-10 and IL-12 [61,65,66]. Inflammatory cytokines have the ability to penetrate the blood–brain barrier and activate microglia, which in turn increases the activity of the enzyme indoleamine-2,3-dioxygenase (IDO), which catabolises tryptophan to kynurenine (KYN) [22,67,68]. The serotonergic transmission is disturbed, and kynurenine, due to its neurotoxic properties, leads to the processes of neurodegeneration of the frontal lobes, hippocampus and amygdala [44,69,70]. The alteration of the kynurenine pathway with the dysregulation of the HPA axis leads to an increase in extracellular glutamate levels and the neurotransmission of glutamate, affecting the neurogenesis of the hippocampus [45,48,71]. It is suggested that this pathophysiological cascade is triggered or sustained and enhanced by chronic inflammation with increased levels of circulating inflammatory markers that are capable of activating microglia and exacerbating inflammation in the nervous system and in consequence leading to the possible disclosure of depressive disorders [34,45,72]. Furthermore, there are studies indicating that the severity of neuroinflammation in the prefrontal cortex, anterior cingulate cortex and insula is associated with the severity of a depressive episode by assessing the expression of translocation protein 18 kDa (TSPO) in positron emission tomography (PET) study [73,74,75]. TSPO is considered a specific biomarker of brain inflammation, particularly due to its high expression in brain immune cells, including activated microglia and stimulated astrocytes, and it can be quantified in PET [73,75]. In addition, Richards et al. show a correlation of IL-5 levels in cerebrospinal fluid, with TSPO binding on PET scan in areas of the subcallosal prefrontal cortex (sgPFC) and anterior cingulate cortex, proving an inflammatory background of depression [73].

## 4. Inflammation in a SARS-CoV-2 Infection

### 4.1. The Renin–Angiotensin–Aldosterone System and the Role of ACE2 

The renin–angiotensin–aldosterone system (RAS) plays an important role in the pathophysiology of a SARS-CoV-2 infection [76]. RAS is responsible for the regulation of the circulating blood volume in the body and the concentration of potassium and sodium ions in body fluids [77]. Furthermore, it is also involved in the pathogenesis of many diseases, such as hypertension, cardiac hypertrophy and fibrosis, atherosclerosis, diabetic micro- and macroangiopathy and inflamed and fibrotic kidneys, as well as in immune disorders [78,79]. The decrease in renal blood flow causes the secretion of renin, which converts angiotensinogen to angiotensin 1 (Ang1), which is then converted by angiotensin-converting enzyme (ACE) to angiotensin 2 (Ang2). The effect of an excessive amount of angiotensin 2 is an increase in vasoconstriction and vascular permeability, activation of cellular pathways and the release of proinflammatory factors including prostaglandins, vascular endothelial growth factor (VEGF), nuclear factor kappa B (NF-κB), TNF-α, interleukin-1β (IL-1β), IL-6 and IFNγ. The SARS-CoV-2 virus enters the cell by binding the S-spike virus to angiotensin-converting enzyme 2 (ACE2) as a specific receptor in the form of a transmembrane protein. ACE2 is widely expressed on the surface of many organ cells, especially in the lungs and intestine, as well as in the cardiovascular system, kidneys, adipose tissue and central nervous system. That is why we observe a range of symptoms in the course of COVID-19, from the symptoms typical of the respiratory and digestive system, to thrombotic events, kidney failure and a number of neuropsychiatric symptoms [80,81]. The appropriate level of ACE2 is necessary for the proper maintenance of homeostasis in cells. ACE2 is responsible for the conversion of angiotensin 2 to angiotensin (Ang) (1–7), which by binding to the Mas receptor (MasR) and angiotensin AT2 receptors (AT2R) produces the opposite effect to angiotensin 2. The ACE2/Ang1–7/MasR axis causes vasorelaxation and suppresses inflammation, oxidative stress, apoptosis, fibrosis and coagulation. Blocking ACE2 by SARS-CoV-2 and down-regulating ACE2 expression leads to the accumulation of angiotensin 2 in the cytoplasm of cells, with a predominance of the pressor arm of the renin–angiotensin system (RAS) [82,83].

### 4.2. Pathophysiology of SARS-CoV-2 Infection

**Figure 2:**
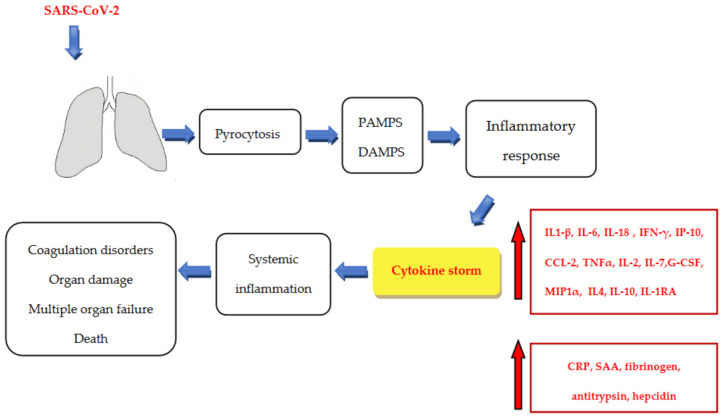
Pathophysiology of SARS-CoV-2 infection. SARS-CoV-2 first infects alveolar epithelial cells, replicates and then induces cell death via a pyrocytosis mechanism causing the release of damage-associated molecular structures (DAMPs) and pathogen-associated molecular patterns (PAMPs) that are recognised by receptors (Toll-like receptors-TLR-s) of neighbouring epithelial cells, endothelial cells and macrophages. The activation of the innate and adaptive inflammatory response results in the release of inflammatory cytokines and chemokines, including IL1-β, IL-6, IL-18, IFN-γ, IP-10, CCL-2, TNF-α, IL -2, IL-7, G-CSF and MIP1α, in addition to IL4, IL-10 and IL-1RA with anti-inflammatory properties. In addition, the level of CRP, SAA, fibrinogen, antitrypsin, hepcidin and complement components that worsen inflammatory reactions and activate the coagulation pathway leading to coagulation disorders increases. As a result of a cytokine storm, systemic inflammation occurs and, as a consequence, organ damage and failure can lead to death. Abbreviations: IL1-β, interleukin 1 beta; IL-6, interleukin 6; IL-18, interleukin 18; IFN-γ, interferon gamma; IP-10, interferon gamma-induced protein 10; CCL-2, the chemokine ligand 2; TNF-α, tumour necrosis factor alpha; IL-2, interleukin 2; IL-7, interleukin 7; G-CSF, granulocyte colony-stimulating factor; MIP1α, macrophage inflammatory protein 1α; IL4, interleukin 4; IL-10, interleukin 10; IL-1RA, interleukin-1 receptor antagonist; CRP, C-reactive protein; SAA, serum amyloid A.

SARS-CoV-2 first infects alveolar epithelial cells, replicates and then induces cell death via a pyrocytosis mechanism causing the release of damage-associated molecular structures (DAMPs) and pathogen-associated molecular patterns (PAMPs) that are recognised by receptors (Toll-like receptors-TLR-s) of neighbouring epithelial cells, endothelial cells and macrophages [84,85] (Figure 2). The activation of the innate and adaptive inflammatory response occurs with inflammatory responses (NF-κB, IL1-β, IL-18 activation) and pro-inflammatory secretion. There is a release of inflammatory cytokines and chemokines typical of T-helper-1 (Th-1), such as IL-6, IFNγ, interferon gamma-induced protein 10 (IP-10) and the chemokine ligand 2 (CCL2), which then attracts and activates monocytes, macrophages and T cells with the aggravation of inflammation at the site of infection. Furthermore, there is an overproduction of TNF-α, IL-2, IL-7, granulocyte colony-stimulating factor (G-CSF) and macrophage inflammatory protein 1α (MIP1α) [18,86,87]. Moreover, there is an increase in T-helper-2 (Th-2) cytokines, including interleukin-1 receptor antagonist (IL-1RA), IL4 and IL-10, with anti-inflammatory effects [86,88,89,90]. IL-6 has a pleiotropic effect and is involved in immune regulation and inflammatory response by inducing various acute phase proteins, such as CRP, SAA, fibrinogen, antitrypsin, hepcidin and complement components that worsen the inflammatory reactions and activate the coagulation pathway leading to coagulation disorders [91,92,93]. When there is inadequate, uncontrolled over-release of cytokines (especially IL-6, IL-1β, TNF-α), a specific "cytokine storm" may occur, with the spread of systemic cytokines and chemokines causing inflammation with over the activation of neutrophils and organ damage leading to multiple organ dysfunction/failure characteristic of high mortality [84,94].

### 4.3. Laboratory Findings

**Figure 3:**
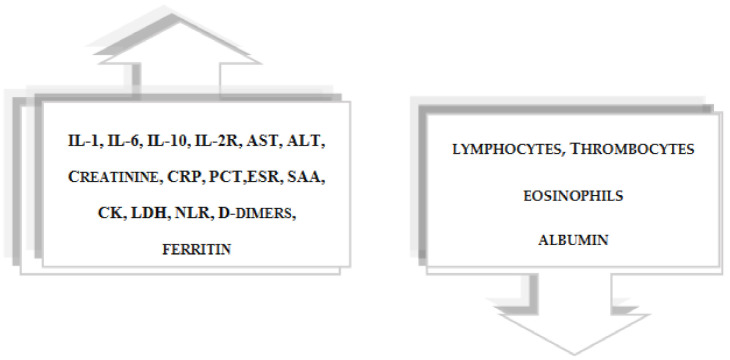
Changes in laboratory parameters in a SARS-CoV-2 infection. COVID-19 infection may be manifested by elevated levels of interleukins (IL-1, IL-6, IL10, IL-2R) and other inflammatory markers (CRP, PCT, ESR, ferritin, SAA) and by liver (AST, ALT) and kidney (creatinine) parameters, as well as by CK, LDH, NLR, D-dimers, correlating with inflammation and decreased levels of lymphocytes, thrombocytes, eosinophils and albumin, depending on the course of infection. Abbreviations: IL-1, interleukin-1; IL-6, interleukin-6; IL-10, interleukin-10; IL-2R, the interleukin-2 receptor; CRP, C-reactive protein; PCT, procalcitonin; ESR, erythrocyte sedimentation rate; SAA, serum amyloid A; AST, aspartate aminotransferase; ALT, alanine aminotransferase; CK, creatine kinase; LDH, lactate dehydrogenase; NLR, neutrophil-to-lymphocyte ratio.

The severity of COVID-19 is correlated with a high level of interleukins IL-6 and IL-1 and with CRP, D-dimers, aspartate aminotransferase (AST), alanine aminotransferase (ALT), creatine kinase (CK), lactate dehydrogenase (LDH), creatinine, low albumin, high erythrocyte sedimentation rate (ESR), low eosinophils, thrombocytopenia and lymphopenia [95,96]. Elevated LDH levels are associated with cell damage. In approximately 80% of patients, lymphopenia and an increased ratio of neutrophils to lymphocytes are observed [16,18,97,98]. Mahat et al. in their review and meta-analysis concluded that in patients with severe COVID-19, serum levels of CRP, ESR, PCT (procalcitonin), IL-6, IL-10, IL-2R, ferritin, SAA (serum amyloid A) and NLR (neutrophil-to-lymphocyte ratio) are significantly increased compared with people with a mild COVID-19. Moreover, they showed increased levels of CRP, PCT, IL-6, ferritin and NLR in non-survivors compared with survivors [99]. Elevated levels of interleukin 6 are characteristic of COVID-19 patients with poor outcomes and are one of the best laboratory indicators of respiratory failure and death. [100,101] IL-6 is considered to be the most significant cytokine in COVID-19, and its increased concentration was also detected in the course of SARS and MERS [102,103]. Elevated serum amyloid A (SAA) levels and disturbances in other biochemical parameters relevant to the development of Alzheimer’s disease have been reported among SARS-CoV-2-positive individuals [99,104,105] (Figure 3). Moreover, the presence of antiphospholipid autoantibodies, which contribute to coagulopathy and ischemic changes in the brain, was observed among COVID-19 patients [24,84,106,107].

### 4.4. The Specificity of Inflammation in COVID-19

SARS-CoV-2 virus induces heterogeneous immune responses [108]. Some have a lack of or mild immune system response, others develop a strong immune response with a cytokine storm and multi-organ damage, including damage to the brain [86,96,109]. The autoimmune response can be induced by a "molecular mimicry" mechanism, whereby autoimmune cells cross-react with autoantigens [15,110,111]. Research suggests that acute SARS-CoV-2 infection causes a subclinical damage accumulation that predisposes to chronic pro-inflammatory disease and may impair the ability to take full advantage of a strong immune system response to infection or trauma. Infected cells persist for up to 6 weeks from the onset of symptoms, and inflammation may persist for weeks after infection has stopped, suggesting an inadequate, excessive inflammatory response from COVID-19, contributing to multiple organ failure [112]. In response to many stressors, such as oxidative stress, metabolic derangement, altered proteostasis, genome instability, macromolecular damage and many others, cell senescence accumulates, which is involved in the pathogenesis of sustained inflammation of the organism [18]. There is a hypothesis that SARS-CoV-2 may influence cellular ageing by causing excessive oxidative stress, DNA damage and metabolic derangement, as well as resulting in a direct effect of viral infection on tissue damage with activation of inflammation [18]. Additionally, overlapping bacterial infections can exhaust the immune response, with physiological dysregulation contributing to immunoageing [18]. As a result of SARS-CoV-2 infection, innate and adaptive immunity may be exhausted. A hallmark of cellular ageing is the release into the blood of senescence-associated secretory phenotype (SASP) factors, such as cytokines and chemokines, which sustain inflammation [113]. Persistent systemic inflammation due to tissue damage, environmental stressors and psychological and social stresses all influence the risk of developing many chronic diseases, including autoimmune diseases, depression, neurodegenerative disorders, accelerated cognitive decline and dementia [107,114].

#### 4.4.1. Neuroinflammation in COVID-19

The immune system constantly communicates with the brain and spinal cord. Even in the absence of damage to the blood–brain barrier, the T- and B-lymphocytes effectors can penetrate the CNS to destroy pathogens [86,109,115,116]. Neuroinflammation is common in various CNS disease states and is characterised by microglia activation accompanied by leukocyte infiltration [117]. Microglia is highly sensitive to changes in peripheral metabolism and comorbidities as well as to external environmental influences, such as stress, diet, physical activity and environmental pollution [107,118]. Glial cells function as resident cells of the innate immune system response to clearing pathogens and damaged brain tissue, and astrocytes play a role in regulating microcirculation in the brain and regulating extracellular glutamate levels [119]. Astrocytes and microglial cells are responsible for maintaining homeostasis, including neurogenesis, synaptic formation, blood–brain barrier control, capture of reactive oxygen species, neurotransmitter uptake, ion transport and blood flow regulation [107,120,121]. Inflammation may have neurogenesis-regulating properties depending on duration and activity of microglia, astrocytes and macrophages. Proteins CD 47, CD 55 and CD 200 as well as interleukin 4 and 10 secreted by stimulated microglia have proneurogenic properties [122]. However, long-term inflammation, through the activation of microglia, increases the concentration of interleukins IL-6, IL-1β and IL-1α and TNF that are unfavourable for neurogenesis [48,123]. As a result of SARS-CoV-2 infection, brain cells, including neurons, oligodendrocytes and glial cells, may lose their physiological functions, leading to a disturbance of homeostasis in the brain. Even after a SARS-CoV-2 infection has ceased, microglia may remain excited indirectly through epigenetic changes [107,124]. Microglial dysfunction, including disorders of neuronal plasticity, synaptic function, myelination and the blood–brain barrier (BBB) maintenance, can severely impair cognitive function, which may have consequences in the short-term and long-term neuropsychiatric consequences of COVID-19 [125]. It is concluded that disturbances at the cellular and molecular level of microglia and other brain cells, along with the accompanying pandemic stress and anxiety, may contribute to the disclosure of mental disorders, including depressive disorders, psychosis and cognitive disorders such as Alzheimer’s disease, Parkinson’s disease and dementia [20,30,107].

#### 4.4.2. Routes of Infection

There are several routes of infection of the nervous system with SARS-CoV-2. The SARS-CoV-2 virus can infect directly by binding to the ACE2 receptor on endothelial [126], nerve and glial cells [127,128,129,130]. In addition, it can penetrate the central nervous system (CNS) through transnasal and transsynaptic invasions [131,132] to the olfactory bulb and then the brainstem, where it can damage the respiratory centres [107,132].

#### 4.4.3. The Effects of Neuroinflammation

As a result of systemic inflammation, circulating cytokines and chemokines can damage the blood–brain barrier and then the brain parenchyma, leading to neuroinflammation with haemorrhage, leukocyte infiltration and neurodegeneration [19,20]. In severe COVID cases, an increase in blood proinflammatory factors such as a “cytokine storm” results in an effect on the CNS and the manifestation of neuropsychiatric symptoms. [23,28,90,133,134]. Nervous system damage manifests itself through the neurological symptoms of COVID-19, such as acute ischemic stroke, meningitis, encephalopathy and Guillain-Barre syndrome as well as psychiatric disorders, including depressive disorders, delirium and psychosis [12,13,30,135]. A number of neuropsychiatric symptoms are observed in the course of COVID-19, and research also shows the occurrence of neurological and psychiatric complications after the time of infection [136]. The studies conducted so far show the occurrence of symptoms of disturbances of consciousness and deterioration of mental state in approximately 20–30% of patients with severe COVID-19 using neuroimaging with MRI [96,137]. Moreover, in one-third of patients with acute/subacute COVID-19 referred for brain imaging, the results showed hypodense/hyperintense areas on MRI/CT and other abnormalities, such as haemorrhagic lesions, including infarcts [138]. In the study by Coolen et al. postmortem magnetic resonance imaging (MRI) results in non-survivor COVID-19 patients showed in approximately 20% olfactory bulb asymmetry and brain parenchyma abnormalities, including micro- and macro-bleeding and oedema changes [139]. An autopsy study of 21 patients with fatal COVID-19 also revealed extensive inflammation in multiple organs, with no SARS-CoV-2 present in any affected organs. In the examined brain tissue, extensive neutrophilic infiltrates with aggregates of NETs (neutrophil extracellular traps) and platelets were found, without the presence of a virus. The mainly affected areas included the olfactory bulb and medulla oblongata, which explains the symptoms of anosmia and the possible increase in hypoxia with respiratory failure during the disease [112].

## 5. The COVID-19 Pandemic as a Psychological Stress Factor

The global transmission of the SARS-CoV-2 virus, the lack of reliable information and disinformation fuelled by sensational headlines in the media heightened fears and social phobias [8,9,140]. A state of nationwide quarantine and additional restrictions were introduced, limiting interpersonal contacts and movement [7,141]. There was fear of uncertainty about the course of COVID-19 disease, the timing of the vaccine, the health service’s ability to fight COVID-19 and government procedures to stop the virus from spreading. In addition, there was a strong stigmatisation of people infected with COVID-19 who unknowingly transmitted the infection to other people, observing symptoms of acute stress disorder, self-destructive behaviour and suicide among them [142,143]. There was widespread anxiety and other psychological problems, including the stigmatisation of people infected with the coronavirus in society, especially among medical staff [144,145]. A survey of 2200 Americans, at a time when the number of confirmed COVID-19 cases in the US was 5, with no fatalities, found that 37% of those polled were worried and concerned about the new SARS-CoV-2 virus, and 25% were more concerned in connection with the COVID-19 pandemic than the Ebola outbreak in 2004 [7]. With the exception of the immediate threat of COVID-19, the psychological impact of the pandemic was observed on the mental health of the general population, including tendencies to closely observe the functioning of the body—analysing cough, shortness of breath and constantly monitoring body temperature—and the appearance of mental disorders with a state of anxiety and panic and obsessive-compulsive disorders in connection with justified recommendations for washing and disinfecting hands [146,147,148]. Psychotic exacerbations or even psychosis have been reported among previously mentally unstable people who succumbed to disinformation during the pandemic [149,150]. The impact of strong anxiety accompanying the pandemic on the aggravation of the pre-existing or the emergence of new mental disorders was observed in susceptible people, deprived of natural defence mechanisms due to the lack of social support and suffering from other mental and somatic disorders and previous traumas, without access to reliable information [142,151,152,153]. Nonetheless, psychiatric disorders are a risk with severe COVID-19. A systematic review of studies, including 43,938 COVID-19 patients with comorbid psychiatric disorders, found that the presence of any psychiatric disorder (especially psychotic disorders and mood disorders) and exposure to anti-anxiety and antipsychotic medications were associated with an increased risk of hospitalisation and a higher risk of COVID-19 mortality. This association also applied, to a lesser extent, to intellectual disability, developmental disorders and substance use disorders, but not to anxiety disorders [154].

Furthermore, the impact of the COVID-19 pandemic and its associated social restrictions has been observed in the progression of cognitive impairment in people with dementia and in the exacerbation of neuropsychiatric symptoms in people with chronic neurological diseases [155]. One study examining the effects of quarantine in patients with Alzheimer’s disease (AD), frontotemporal dementia (FTD), vascular dementia (VD) and dementia with Lewy bodies (DLB) found that approximately 60% of patients experienced changes in behavioural and psychological symptoms (BPSD) after one month of isolation, including worsening of previous symptoms (51.9%) or emergence of new symptoms (26%). The most commonly reported symptoms that worsened were irritability, agitation, apathy, depression, anxiety and sleep disturbances. Depending on the type of dementia, severity of prevalence and gender of patients, differences in the trend of reported symptoms were observed. Symptoms of anxiety and depression were more typical for the female gender, patients with AD and mild to moderate disease course. In addition, DLB was associated with a higher risk of increased sleep disturbances and hallucinations as well as FTD with changes in appetite [156]. Another cross-sectional case-control study to quantify anxiety in Parkinson’s disease (PD) patients in relation to social distance restrictions compared with the general population showed the highest levels of anxiety in the PD group [157]. It is suggested that anxiety in PD patients is strongly correlated with the risk of a COVID-19 infection, the availability of the drug in a blockade situation and the perceived higher risk of disease due to chronic comorbidity [157]. In addition, a COVID-19 infection may contribute to the progression of neurodegenerative diseases, such as amyotrophic lateral sclerosis, by exacerbating inflammation, and the progression of the disease state may negatively affect patients’ psychological status. [158]. Outbreaks of pandemics were invariably associated with panic states and a sense of threat to individual safety due to high mortality. Widespread health effects of the pandemic have been reported, including anxiety, insomnia, increased alcohol consumption and loss of energy [159]. Studies on the observed psychological reactions of society during previous pandemics suggest that the response to stress may be influenced by individually diverse factors, such as individual intolerance to uncertainty, perception of one’s own susceptibility to illness and tendency to anxiety [142,160]. One study found that the "vulnerable group" (quarantined or relatives or suspected/ill persons) experienced depressive symptoms more frequently during the SARS pandemic compared with the "non-exposed group". Other studies have found that more than 40% of people who developed SARS experienced symptoms of post-traumatic stress disorder (PTSD) at some point during the pandemic. The respondents who were isolated worked in places with a high risk of SARS infection—e.g., individuals in infectious wards or relatives of patients who experienced contact with SARS were 2–3 times more predisposed to developing severe PTSD symptoms compared with people not exposed to the virus [161]. The above studies and the psychological impact of previous pandemics demonstrate the possible impact of the larger global COVID-19 pandemic on the increase in the incidence of PTSD in the population [162,163,164].

## 6. Depressive and Neurocognitive Disorders during a COVID-19 Pandemic

The outbreak of a pandemic and rapid changes in almost every aspect of life, social isolation, quarantines and uncertainty about the future can be treated as strong stressors dysfunctionally affecting mental health [165,166]. Studies have shown that COVID-19 patients are at high risk of stress and feelings of stigma [167,168] and are more likely than the general population to suffer from a variety of mental disorders, including depression, anxiety disorders, acute stress disorder, post-traumatic symptoms and insomnia [26,169]. An analysis of COVID-19 patients showed that 1 in 3 presented executive dysfunction and problems with attention and orientation, and areas of hypoperfusion were observed in brain imaging studies [170]. A study of survivors of COVID-19 conducted in Wuhan 6 months after recovery showed symptoms of depression among 23% of patients (367 out of 1617) [171]. One of the prospective studies concerned patients with SARS-CoV-2 admitted to the non-intensive ward, who during hospitalisation showed symptoms of depression in 29% of the patients, while 2 weeks after discharge, in 20% of the patients, symptoms of depression were still observed. This may indicate a relatively stable severity of depression in the subjects shortly after being infected with COVID-19 [172]. Moreover, one of the studies showed that approximately 1 month after infection 31–38% of patients reported symptoms of depression [173]. Another prospective study assessing the mental state and the level of inflammatory markers in COVID-19 survivors one month and three months after discharge from the hospital showed a relationship between inflammation after COVID-19 and depression and related neurocognitive disorders. The 3-month follow-up cohort showed disorders in at least one psychopathological sphere in 35.8% of respondents. Moreover, there was a tendency to intensify depressive symptoms, especially in people with a previous history of mental disorders and in the females, as well as an association of acute infection with the manifestation of neurocognitive disorders including motor coordination and executive dysfunctions in up to 78% of the respondents. With observation, symptoms of depression persisted in contrast with symptoms of anxiety, PTSD and sleep disorders, which improved over time [19]. COVID-19 may develop prolonged inflammation, predisposing to persistent depression and related neurocognitive disorders [18,20,22,24,174]. It is associated with the SARS-CoV-2 infection itself inducing a specific inflammatory response with possible long-term elevated levels of inflammatory markers [18,19,175,176]. In a study from Ireland, approximately 25.3% of 150 patients with COVID-19 had elevated D-dimers 4 months after diagnosis, which was more common in patients requiring hospitalisation and those who were over 50 years of age [177]. A previous study of COVID-19 survivors indicates a relationship between cognitive dysfunction in maintaining attention and underlying inflammation as measured by CRP [125]. Moreover, the baseline level of SI (II) systemic immune inflammation associated with the level of lymphocytes, neutrophils and platelets was found to be related to the severity of depression symptoms and the prediction of neurocognitive impairments [19,178]. In a study by Zhou et al., higher levels of SI (II), which can be considered a marker of the low-grade inflammation observed in a mood disorder [23], have been associated with a major depressive disorder [179]. In one meta-analysis presenting results mainly from China, up to 45% of COVID-19 patients were shown to experience depression, with no gender differences found. However, other studies show that women, people with a severe history of COVID-19, those with elevated markers of inflammation, people with infected family members and patients with an earlier psychiatric diagnosis are in the risk group of developing depressive disorders [178,180].

### Post-COVID Syndrome

**Figure 4:**
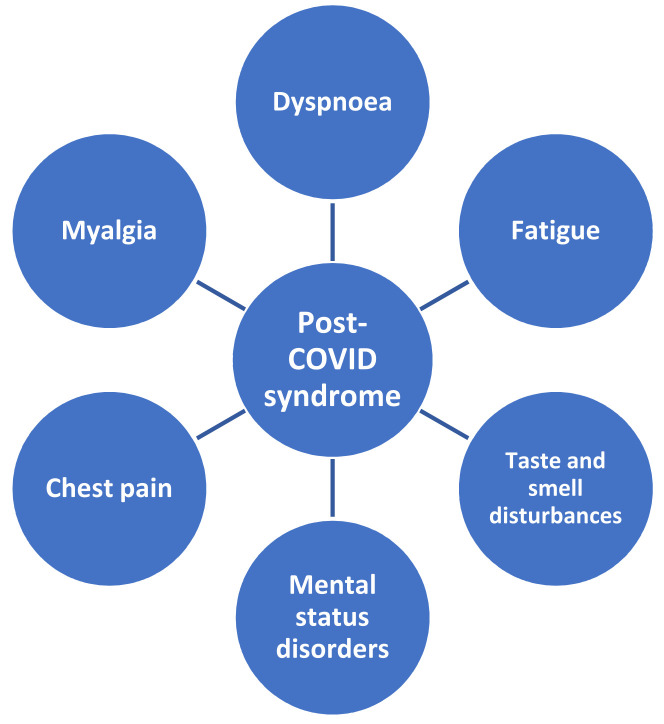
Symptoms of Post-COVID syndrome.

Symptoms following COVID-19 infection may persist for up to several weeks after an acute infection. Patients with symptoms that persist more than 3 weeks after diagnosis are said to have post-COVID syndrome [181,182]. The syndrome has been suggested to result from prolonged inflammation following a SARS-CoV-2 infection, although the pathogenesis is still under investigation and is not entirely clear. Characteristic symptoms of this syndrome include dyspnoea, chest pain, myalgia, fatigue, and taste and smell disturbances, as well as mental status disorders (Figure 4) [176,183,184].

One study identified the top three symptoms of post-COVID syndrome, which included fatigue, cognitive dysfunction and general malaise [185]. The prospective observational study of patients hospitalised with COVID-19 showed that 4 months after discharge, 51% (244 of 478) had at least one symptom that was not present before the illness, including (31%) reported fatigue, (21%) cognitive symptoms and dyspnoea [184]. In non-hospitalised patients after COVID-19, without comorbidities, it is estimated that 10% to 35% may present with symptoms of post-COVID syndrome [183,186,187], and in hospitalised patients with severe COVID-19, the incidence is up to 80% [182,188,189]. An Italian study of 238 patients hospitalised with severe COVID-19, 4 months after discharge, showed prolonged pulmonary dysfunction in 53.8%, which may account for many post-COVID symptoms [190]. It seems important to follow up on patients, especially in the area of cognitive function, where neurological and cerebrovascular complications were observed during the course of the disease [19,191,192,193]. Direct infection of endothelial cells by SARS-CoV-2 as well as general inflammation contribute to coagulopathy and embolic/thrombotic complications, microcirculatory disorders affecting hypoxia and other symptoms in post-COVID syndrome [136,182,193]. Neurological and cerebrovascular complications are characteristic, although they are less common and may be implicated in the development of Alzheimer’s disease [194]. In addition, it was indicated that in all patients after COVID-19, compared with controls, protein markers of neuronal dysfunction such as amyloid beta, total tau and p-T181, neurogranin and neurofilament light protein were increased in neuron-enriched extracellular vesicles (nEV), indicating an association with neuroinflammation and neurodegeneration [195]. Furthermore, the genetic risk factor of Alzheimer’s disease (ApoE4) has been shown to increase the risk of severe COVID-19 infection, but it is still unclear [196]. It has been reported that 23 of 27 patients with Parkinson’s disease and COVID-99 developed post-COVID syndrome, deterioration of motor function, increased fatigability and increased need for levodopa, as well as sleep and cognitive dysfunction [197]. In one prospective UK study, at least 4 weeks after recovery from acute COVID-19 infection, MRI in more than 70% of patients showed dysfunction in at least one organ, indicating a physiological basis for the infection and possible long-term impairment of body organs [198]. Organ failure was related to heart (systolic dysfunction, myocarditis), liver (hepatomegaly, inflammation, ectopic fat), lung (decreased vital capacity), kidney (inflammation), pancreas (inflammation, ectopic fat) and spleen (splenomegaly). Furthermore, of the incidental more severe structural MRI lesions (n = 56), three were cardiac lesions, and one was renal (hydronephrosis) [198]. Brain MRI findings of a 56-year-old previously healthy patient with neurological symptoms and depression almost 6 months after a COVID-19 infection showed multiple hyperintense areas in the white matter and semi left centres, indicating neurodegeneration and micro-vascular damage [199]. One study of plasma from patients after COVID-19 at 1–3 months found elevated levels of IL-4, which may indicate ongoing neuroinflammation [195]. Another study of 12 patients with neurological complications presenting with post-COVID-19 symptoms between 9 and 12 weeks post-onset found an acute inflammatory phase with significantly elevated inflammatory parameters including C-reactive protein CRP and IL-6 levels [30]. Elevated inflammatory markers may, by impairing the blood–brain barrier, contribute to neuropsychiatric complications including neurocognitive complications through alterations in neurotransmission, including gamma-aminobutyric acid (GABA) [200]. Previous studies have shown in animal models that inflammation with elevated IL-6 levels can decrease GABA receptor density [200]. Furthermore, compared with healthy individuals, evidence of altered neuronal function, neuromotor fatigue, impaired cognitive control, executive dysfunction and apathy was found in patients with post-COVID syndrome [201]. Mental status disorders, including depressive disorders, may affect 26% to 40% of patients even up to 6 months after the onset of symptoms [171,174]. In addition, based on two cases of patients with depression after COVID-19, an association between depression and interleukins, including IL-6, was demonstrated independent of other factors, which may justify the administration of cytokine-reducing drugs to prevent depression after COVID-19 [175]. Moreover, 8 patients with neurological symptoms, compared with 16 without neurological symptoms, had higher levels of anti-SARS-CoV-2 antibodies with an increase in IL-6 [176].

## 7. Antidepressant Treatment and COVID-19

Depression is associated with low-grade chronic inflammation and is comparable with a chronic cold [44,202]. Patients with chronic inflammatory processes and autoimmune diseases are more prone to depression [43,54,60]. In addition, the assessment of the concentrations of small molecules—metabolites that are products of changes taking place in the body—allows indirect conclusions to be made about the disturbances of specific metabolic pathways [34,203]. The antidepressant treatment used in depressive disorders has antioxidant and anti-inflammatory properties, resulting in a reduction in inflammatory cytokines including CRP and IL-6, [204], with an increase in the concentration of anti-inflammatory cytokines [205,206]. This is supported by neuroimaging studies using PET, showing less neuroinflammation in treated depressed patients compared with untreated patients, which may suggest the normalisation of TSPO expression in the brain and inhibition of microglia activity as a result of antidepressant treatment [73,207]. One study demonstrated a significant effect of antidepressant treatment as well as its duration on the total volume distribution of TSPO in patients with a history of major depressive disorder. In patients untreated for major depressive disorder for 10 years or more, TSPO volume distribution was up to 33% greater in the anterior cingulate cortex, prefrontal cortex and insula compared with participants untreated 9 years or less [208]. It is expected that, in people with a history of COVID-19, the concentration of inflammatory parameters may be higher and the response to antidepressant treatment less effective due to the possible prolonged, abnormal inflammation, with lesions at the level of the nervous system following SARS-CoV-2 infection. Differences in cytokine concentrations in patients may predict disease or resistance to treatment. Based on the current medical knowledge, it is suggested that a history of a SARS-CoV-2 infection may have a significant impact on the reduction in neurocognitive functions [19,125,174,194]. Due to its anti-inflammatory properties, anti-depressant treatment may reduce the inflammatory parameters [209,210]. However, the use of antidepressants to improve neurocognitive impairment is questionable. There are studies indicating the progression of cognitive dysfunction after the inclusion of antidepressants [211,212]. Previous knowledge indicates that, among the selective serotonin reuptake inhibitors (SSRIs) group antidepressants, fluoxetine is a promising drug against COVID-19 through its effect on reducing the secretion of inflammatory cytokines/chemokines (IL-6,CCL-2,TNF-α) and immunomodulatory properties [32,209,210]. In addition, fluoxetine exhibits antiviral activity and is effective against SARS-CoV-2 in cell cultures [32]. This indicates the possible alleviation of neuropsychiatric complications after COVID-19 by fluoxetine use [32]. Such a potent inhibitory effect in both pseudovirus and authentic virus assay has also been described for vortioxetine [213].

## 8. Discussion

In this review, we present the current knowledge regarding the possible manifestation of depressive and neurocognitive disorders due to the inflammatory background of COVID-19. The experience of previous pandemics, including SARS-CoV, MERS, influenza of the 18th and 19th centuries and Spanish flu in the 20th century, indicates that there is an impact on the development of mental disorders with cognitive deterioration even months after the onset of the disease [18,214]. Studies of convalescents after infection with SARS-CoV and MERS coronaviruses indicate that up to 15% of respondents reported memory, attention and concentration disorders lasting from 6 weeks to 39 months from the onset of the disease [28,29].

Increasingly more studies confirm the hypothesis that inflammation caused by SARS-CoV-2 infection may, in the short term [11,19,178] and long term [2,215], have negative health consequences [182,199,216], including in the field of mental health disorders [18,148,169]. Patients who are severely affected by COVID-19 with respiratory symptoms leaving intensive care units are potentially more likely to experience long-term neuropsychiatric and neurocognitive conditions, such as depression, obsessive-compulsive disorder, psychosis and Parkinson’s and Alzheimer’s disease [20,30]. Studies show the correlation of inflammation with the severity of complications of post-COVID-19 syndrome, including neuropsychiatric disorders [20,30]. Studies of convalescents one month and 3 months after discharge from the hospital indicate an increase in the incidence of psychiatric disorders, including depressive disorders and a deterioration of cognitive functions [28,30,105,217]. In addition, stress during a pandemic, associated with a change in life in almost every aspect, fear of illness, death and other psychosocial factors [7,49,165], can activate the stress axis [46], affect the severity of inflammation in the brain and, consequently, its structural and functional changes [55,65,71,218]. Research confirms that females, the healthcare professionals, elderly individuals, children, college students and psychiatric patients are in the group at increased risk of developing depressive disorders during the COVID-19 pandemic [151,219,220,221,222,223].

An increasing number of studies of COVID-19 survivors show elevated inflammatory markers, including interleukins and CRP, indicating persistent inflammation in the body months after infection [30,175,176,184]. An interesting study found elevated parameters of D-dimers and fibrin products that may be increased during ongoing inflammation and which were observed 6 months after infection with COVID-19 [177]. In addition, observed multisystem inflammatory syndrome in children and adolescents (MIS-C) infection with elevated levels of immunoglobulins, C-reactive protein, ferritin and interleukin-6 indicate possible 4-week post-viral immunisation in the body [224]. Moreover, several cases of adults with MIS-A (multisystem inflammatory syndrome in adults), characterised by a wide spectrum of gastrointestinal, cardiovascular, dermatologic and neurologic symptoms, have been described [225]. As a result of excessive, uncontrolled inflammatory response, immune system dysfunction, including autoimmunity, may occur [18,111]. The presence of antiphospholipid antibodies has been demonstrated in patients with COVID-19, which indicates possible autoimmunity and subsequent attack of the body’s own cells and a number of other complications, including a tendency to thrombosis [106,107].

The analysis of COVID-19 patients showed that every one-third of them presented with executive dysfunction with attention and orientation problems, and areas of hypoperfusion were observed in brain imaging studies [170]. There is evidence that brain hypoperfusion may accelerate amyloid-β (Aβ) accumulation, the pathology of the tau and TDP-43 proteins involved in the development of dementia [105,194]. The white matter of the brain is also very sensitive to ischemia in COVID-19, which affects cognition [194].

The SARS-CoV-2 virus enters the cell by binding ACE2, which is widely expressed on the surface of glial cells, nerve cells and endothelial cells [107]. That is why we observe a number of neuropsychiatric symptoms [12,13,137,150]. Moreover, microglia may remain in an activated state even after cessation of the infection, causing neurotransmission disorders and structural changes in the brain affecting the manifestation of neurocognitive and depressive disorders over time [47,118,120,124,218,226,227]. Although some studies do not indicate the presence of virus in the brain in autopsy studies, the existing neuroinflammation may be the result of a strong systemic inflammatory response damaging the blood–brain barrier [112]. 

It has been suggested that the COVID-19-related cause of death may be induced by a specific “cytokine storm” with an elevated level of cytokines, (especially IL-1 and IL-6, systemic inflammatory response syndrome—SIRS) leading to multiple organ failure with high mortality [14,15,16,84,86,93]. Recent publications have shown that ACE2 expression is higher in males, which is associated with greater susceptibility to SARS-CoV-2 infection compared with females, which also explains the higher male morbidity and mortality rates [83]. The administration of interleukin 1 and 6 inhibitors in seriously ill COVID-19 patients with respiratory failure and hyperinflammation causes a significant reduction in mortality in patients with IL-1 inhibition. Interleukin 6 inhibition was effective in patients with high levels of CRP, and inhibitions of both IL-1 and IL-6 were effective in patients with low levels of lactate dehydrogenase (LDH) [228]. 

There are reports of improvement in post-COVID-19 symptoms due to a vaccine that may improve the immune response or reverse autoimmunity [182,229]. In addition, scientific evidence suggests the efficacy of antidepressant treatment having antiviral and anti-inflammatory properties, especially from the SSRI group of drugs, including fluoxetine [32,209,210,213].

Current evidence suggests that symptoms in post-COVID syndrome improve over time and patients show a good prognosis without further sequelae [230]. However, the duration and long-term effects of post-COVID syndrome are unknown and still need further study [31]. It seems important to observe patients, especially in terms of cognitive functions, in whom neurological and cerebrovascular complications were observed during the disease [138,231,232]. There is still insufficient research to establish the exact relationship between the long-term consequences of COVID-19 and the inflammation it causes [18,19,24,84,233].

## 9. Conclusions

It is increasingly known that, during SARS-CoV-2 infection and after recovery, patients are more likely to develop psychiatric disorders, including depressive and neurocognitive disorders. Further research is required to expand the knowledge on the impact of SARS-CoV-2 infection on the intensification or disclosure of depressive disorders and neurocognitive disorders. Research will broaden our understanding of the possible long-term neuropsychiatric consequences of a COVID-19 infection. At the same time, the studies will help to identify risk groups in terms of difficulties in treating mood disorders, as well as helping to develop potential new therapeutic methods in the future. In addition, the results of the research may increase public awareness of the serious impact of the COVID-19 pandemic on mental health, while paying attention to the need to prevent depression as a serious, potentially life-threatening disease entity. Moreover, it is predicted that, in the coming months and even years, numerous post-COVID-19 patients will seek medical attention from specialists due to complications and symptoms of post-COVID syndrome. It is important to clarify the pathogenesis of SARS-CoV-2 infection and its consequences in the body, including post-COVID-19 syndrome, and to identify markers and targeted therapy. New guidelines are needed for the diagnosis and treatment of this new clinical entity.

## 10. Limitations

The review did not address the aspect of the manifestation of depressive disorders during COVID-19 in specific age groups, including young adults and the elderly as well as patients with pre-existing inflammatory process. Due to the growing number of studies, only the individual and subjectively most significant results were presented to substantiate the association of inflammation after COVID-19 infection with the manifestation of neurocognitive and depressive disorders. No clear-cut, definite theories of the effect of SARS-CoV-2 on long-term complications exist and, therefore, time for observation, research and further analysis is needed.

## Data Availability

Data sharing not applicable.
